# First Isolation and Molecular Characterization of *bla*_**CTX-M-121**_-Producing *Escherichia coli* O157:H7 From Cattle in Xinjiang, China

**DOI:** 10.3389/fvets.2021.574801

**Published:** 2021-05-25

**Authors:** Zhanqiang Su, Panpan Tong, Ling Zhang, Mengmeng Zhang, Dong Wang, Kaiqi Ma, Yi Zhang, Yingyu Liu, Lining Xia, Jinxin Xie

**Affiliations:** College of Veterinary Medicine, Xinjiang Agricultural University, Urumqi, China

**Keywords:** *E. coli* O157:H7, virulence genes, antibiotic resistance, PFGE, bovine

## Abstract

The bovine *Escherichia coli* O157:H7 is a major foodborne pathogen causing severe bloody diarrhea, hemorrhagic colitis, and hemolytic uremic syndrome in humans. Cattle are recognized major reservoir and source of *E. coli* O157:H7. We investigated the antibiotic resistance, molecular profiles, and intrinsic relationship between 21 isolates of *E. coli* O157:H7 from cattle farms and slaughtering houses in Xinjiang. Using pulsed-field gel electrophoresis (PFGE) molecular typing, two types of PFGE were revealed through cluster analysis, including clusters I and II, with 66 and 100% similarity of PFGE spectra between 21 isolates. We also detected that 18 isolates (86%) carried at least one virulence gene, 16 isolates (76%) carried the *eae* gene, and 7 (33%) carried the *stx1* + *stx2* + *eae* + *hly* + *tccp* genes. Eighteen isolates were susceptible to antibiotics. Three isolates were resistant to antibiotics, and two were multidrug resistant. One of the two multidrug-resistant isolates detectably carried the *bla*_CTX−M−121_ gene. This is the first finding of the *bla*_CTX−M−121_ gene detected in *E. coli* O157:H7 isolated from cattle in Xinjiang. The *bla*_CTX−M−121_ gene is transferable between the bacterial strains via plasmid transmission. The results indicated that *E. coli* O157:H7 may have undergone clonal propagation in cattle population and cross-regional transmission in Xinjiang, China.

## Introduction

*Escherichia coli* O157:H7 is a major foodborne pathogen that causes severe bloody diarrhea, hemorrhagic colitis (HC), and hemolytic uremic syndrome (HUS) in humans (1). *E. coli* O157:H7 was first recognized as a pathogen contributing to an outbreak of HC associated with hamburger consumption in 1982 (2). Since then, *E. coli* O157:H7 outbreaks have been reported in the United States, Canada, Japan, and China (3–6). *E. coli* O157:H7 has been reportedly detected in healthy cattle worldwide (7). The infected, asymptomatic cattle irregularly excrete *E. coli* O157:H7, resulting in contaminating food and water in the environment, as well as infecting humans and other animals (8). Cattle are recognized major reservoir and source of *E. coli* O157:H7.

Pathogenic virulence of *E. coli* O157:H7 is attributable to genes coding for Shiga toxin (Stx), the intestinal cell shedding site [locus of entericyte effacement (LEE)] virulence island, and the large plasmid pO157 (9). Stx, comprising Stx1 and Stx2, is able to induce cell necrosis and tissue lesions, and Stx2 is more potent than Stx1 (10, 11). The LEE region encodes a type III secretion system, and the secreted proteins *E. coli* secreted proteins (Esp) and translocated intimin receptor (Tir). Both Esp and Tir are required for intimate attachment and A/E lesion formation (12). The LEE region also encodes intimin, an outer membrane protein adhesin (Eae) that mediates the intimate attachment of bacteria to the host epithelial cell surface (13). In addition, Tir cytoskeleton-coupling protein (TccP) stimulates actin polymerization during the formation of A/E lesion (14). The large plasmid pO157 carries genes coding for type II secretion systems, such as hemolysin (Hly) and ToxB. All these virulence factors of *E. coli* O157:H7 reportedly regulate the adhesion of pathogenic bacteria to intestinal epithelial cells, causing the shedding of intestinal cells. These virulence genes have been used to identify bacterial strains isolated from various sources in epidemiological studies (6, 15, 16).

Antimicrobials have been the mainstay for the prevention and treatment of bacterial diseases in animals. However, their use is getting limited due to rising antibiotic resistance, which has become a serious problem worldwide, especially in developing countries where the quality, distribution, and use of antibiotics in human and veterinary medicine is not strictly regulated (15, 17). Extended-spectrum cephalosporins (ESCs), especially the third- and fourth-generation cephalosporins, are classified by the World Health Organization (WHO) to treat infections of multidrug-resistant Gram-negative bacteria (18). However, acquisition of genes encoding extended spectrum β-lactamases (ESBLs), especially CTX-M enzymes, by *E. coli* plays an important role in the resistance to ESCs (19). The genes encoding these enzymes, i.e., *bla*_CTX−M_ genes, are usually located on transferable plasmids, which also carry resistance genes for other types of antimicrobials (i.e., fluoroquinolones, aminoglycosides). These plasmids mediate the spread of drug resistance between bacteria *via* conjugation (20). *E. coli* O157:H7 isolates collected from humans and animals have shown resistance to a variety of antibiotics; therefore, the emergence of multidrug resistant (MDR) *E. coli* O157:H7 has become a public health issue (21, 22).

The sustainability of cattle industry and food safety depend upon the effective prevention and control of bovine pathogenic microorganisms. Xinjiang is one of largest cattle-raising regions in China. To further assess the potential public health impact of *E. coli* O157:H7 in Xinjiang, we investigated the pathogenicity and antibiotic resistance of isolates collected from farms and slaughterhouses. We examined the intrinsic relationship among different isolates and assessed the potential dissemination of MDR profiles *in vitro*.

## Materials and Methods

### Sample Collection

Total samples (*n* = 2,439) included 1,155 fresh feces, 1,236 rectal swabs, and 48 carcass swabs that were collected from 18 beef cattle and dairy farms (industrial, semi-industrial, and traditional farms, with a herd size of 200–8,000 cattle) and one slaughterhouse in the region of Akesu, Bole, Changji, Tacheng, Urumqi, Wujiaqu, and Yili in Xinjiang, China between October 2012 and March 2017. Samples were collected from Xinjiang brown cattle, Holstein cattle, Simmental cattle, and Angus cattle (1–7 years old, 400–800 kg body weight). Approximately 25 g of fecal samples were collected from each animal by rectal palpation or during defecation using disposable sleeve gloves and then placed in sterile Whirl-Pak bags (Nasco, Fort Atkinson, WI, USA). Rectal swabs were collected when rectal palpation is not applicable or no bowel movement is observed. Sterile cotton swabs (length, 150 mm; Copan Italia SpA) were used to collect mucus samples from the rectal anal junction. Sterile cotton swabs were also used to swab ~10 cm^2^ of carcass surface. All samples were transported in icebox to the laboratory and stored at 4°C until processed within 2 h.

### *Escherichia coli* O157:H7 Isolation

Selective enrichment was carried out according to the method reported by Mersha et al. (23) with minor modifications. One gram of each feces was aseptically added to 9 ml of modified tryptone soya broth containing 20 mg/L novobiocin (mTSB + n) (Hopebio, Qingdao, China) and incubated at 37°C for 16 h. To all the swab samples, 90 ml of mTSB + n was added and homogenized using a vortex mixer. After incubation for 16 h, all the samples were processed for immunomagnetic separation (IMS) using anti-*E. coli* O157 Dynabeads (Dynal, Invitrogen, USA) as follows. One microliters of the enriched broth culture was put in a sterile screw cupped Eppendorf tubes to which 20 μl of anti-O157:H7 immunomagnetic beads was added, followed by shaking at ambient temperature for 30 min. The tubes were then kept inside the manual magnetic particle concentrator. The beads were washed thrice using 300 μl phosphate-buffered saline (PBS) buffer for each wash. Finally, 100 μl of PBS was added in each tube and mixed gently (24). Fifty microliters of the mixture was streaked onto Sorbitol MacConkey agar containing 0.05 mg/L cefixime and 2.5 mg/L potassium tellurite (CT-SMAC) (Hopebio, Qingdao, China) and incubated at 37°C for 20–24 h to develop colonies. Pale-colored colonies were purified by repeated streak plating until a uniform colony morphology was obtained (25). One or more of the colonies were individually selected as presumptive *E. coli* O157 per sample. *E. coli* CICC 21530 (O157:H7, *stx1* + *stx2* + *eae* + *hly* + *tccp*) (26, 27) and ATCC 25922 strains were used as positive and negative controls, respectively. Two genes (*rfbEO157* and *fliCH7*) were used to identify *E. coli* O157:H7 (28). Pink colonies (suspected the general *E. coli*) were purified by restreaking on McConkey agar and confirmed by PCR method as described by Teichmann et al. (29) (Table 1). The PCR amplicons (10 μl) were subjected to electrophoresis on a 1.2% agarose gel in 1× Tris–acetate–EDTA (TAE) buffer at 115 V for 30 min and stained with SYBR Green (Fermentas, Germany). The positive isolates were each inoculated in separate TSB and incubated overnight at 37°C, from which glycerol stocks were made and then stored at −80°C for further analysis.

**Table 1:** Primers used in PCR to detect targeted genes.

**Gene**	**Primer oligonucleotide sequences (5′-3′) (forward/reverse)**	**Amplicon size (bp)**	**Annealing temperature (°C)**	**Reference**
*bla*_CTX−M−U_	ATGTGCAGYACCAGTAARGT/TGGGTRAARTARGTSACCAGA	593	50	(35)
*bla*_CTX−M−1G_	GTTACAATGTGTGAGAAGCAG/CCGTTTCCGCTATTACAAAC	1,018	50	(35)
*bla*_CTX−M−2G_	ATGATGACTCAGAGCATTCG/TGGGTTACGATTTTCGCCGC	865	55	(36)
*bla*_CTX−M−9G_	ATGGTGACAAAGAGAGTGCA/CCCTTCGGCGATGATTCTC	870	60	(37)
*bla*_TEM_	ATGAGTATTCAACATTTCCGT/TTACCAATGCTTAATCAGTGA	861	48	(38)
*bla*_SHV_	CCGGGTTATTCTTATTTGTCGCT/TAGCGTTGCCAGTGCTCG	1,081	48	(39)
*cmlA1*	CCGCCACGGTGTTGTTGTTATC/CACCTTGCCTGCCCATCATTAG	698	59	(39)
*eae*	CATTATGGAACGGCAGAGGT/ACGGATATCGAAGCCATTTG	375	52	(31)
*fliCH7*	TACCATCGCAAAAGCAACTCC/GTCGGCAACGTTAGTGATACC	247	58	(28)
*hly*	CACACGGAGCTTATATTCTGTCA/AATGTTATCCCATTGACATCATTTGACT	319	45	(32)
*rfbEO157*	CTACAGGTGAAGGTGGAATGG/ATTCCTCTCTTTCCTCTGCGG	327	58	(28)
*stx*1	GAAGAGTCCGTGGGATTACG/AGCGATGCAGCTATTAATAA	130	54	(30)
*stx*2	TTAACCACACCCACGGCAGT/GCTCTGGATGCATCTCTGGT	346	54	(30)
*tccP*	CGCCATATGATTAACAATGTTTCTTCAC/CTCGAGTCACGAGCGCTTAGATGTATT	700~1,000	58	(14)
*sul1*	CGGCGTGGGCTACCTGAACG/GCCGATCGCGTGAAGTTCCG	433	65	(40)
*tetA*	GCTACATCCTGCTTGCCTTC/CATAGATCGCCGTGAAGAGG	210	55	(41)
*tetE*	AAACCACATCCTCCATACGC/AAATAGGCCACAACCGTCAG	278	55	(41)
*tetG*	GCTCGGTGGTATCTCTGCTC/AGCAACAGAATCGGGAACAC	468	55	(41)

### Analysis of Virulence Genes

Genomic DNA contents were extracted from 21 *E. coli* O157:H7 isolates as confirmed by PCR serotyping. In brief, 3–5 colonies were individually suspended in 200 μl of sterile distilled water. Bacterial suspensions were then heated at 95°C for 10 min centrifugation at 13,400 × g for 10 min to obtain the supernatant containing the template DNA and were transferred into 1.5-ml Eppendorf tubes without nuclease and stored at −20°C.

A multiplex PCR procedure was used to detect the *stx1* and *stx2* genes (30), and a single PCR procedure was used to detect the *eae* (31), *hly* (32), and *tccp* (14) genes. The primers, conditions, and references cited are listed in Table 1. *E. coli* CICC 21530 was used as a positive control for all the five virulence genes, while ATCC 25922 was used as a negative control. Amplification of the targeted gene was carried out using EX Taq (TaKaRa, Dalian, China) with the following PCR program: 94°C for 4 min, 30 cycles of denaturation at 94°C for 30 s, annealing at 54°C for 30 s, and extension at 72°C for 30 s, with a final extension at 72°C for 10 min. The annealing temperature was adjusted according to the primer Tm value (Table 1).

### Antimicrobial Susceptibility Tests

Antibiotic susceptibility was tested using the Kirby–Bauer disk diffusion technique. Antibiotic disks of 6 mm in diameter obtained from OXOID, UK, containing ampicillin (AMP, 10 μg/disk), piperacillin (PIP, 100 μg/disk), cefotaxime (CTX, 30 μg/disk), ceftazidime (CAZ, 30 μg/disk), cefepime (FEP, 30 μg/disk), aztreonam (ATM, 30 μg/disk), ampicillin-sulbactam (SAM, 10/10 μg/disk), piperacillin-tazobactam (TZP, 100/10 μg/disk), amoxicillin-clavulanic acid (AMC, 20/10 μg/disk), gentamicin (GEN, 10 μg/disk), amikacin (AMI, 30 μg/disk), streptomycin (STR, 10 μg/disk), cotrimoxazole (SXT, 25 μg/disk), chloramphenicol (CHL, 30 μg/disk), levofloxacin (LEV, 5 μg/disk), ciprofloxacin (CIP, 5 μg/disk), tetracycline (TET, 30 μg/disk), and polymyxin B (PB, 300 U/disk) (20). *E. coli* ATCC25922, purchased from China Center of Industrial Culture Collection (CICC), was used as a quality control strain in the susceptibility tests. The ESBL-producing isolates were determined by double-disk synergy tests according to CLSI (33). Isolates shown to be resistant to at least three different classes of antimicrobial agents were determined to be multidrug resistant (MDR) (34).

### Detection of Antibiotic Resistance Genes

The following resistance determinants were investigated by PCR: *bla*_CTX−M_ [the CTX-M-type genes were detected using universal primers *bla*_CTX−M−U_ (35), and the entire CTX-M-type genes were amplified using the primers *bla*_CTX−M−1G_ (35), *bla*_CTX−M−2G_ (36), or *bla*_CTX−M−9G_ (37)], *bla*_TEM_ (38), and *bla*_SHV_ (39), which encode β-lactamases, chloramphenicol efflux pumps [*cmlA1*(39)], sulfonamide resistance gene [*sul1* (40)], and the *tetA* (41), *tetE* (41), and *tetG* (41) tetracycline efflux pumps. *bla*_TEM_ and *bla*_SHV_ genes were amplified by double PCR; *tetA, tetE*, and *tetG* genes were amplified by triplex PCR, while other resistant genes were amplified by single PCR. Primers used for the different genes are listed in (Table 1). The PCR products were sent to Sangon Biotech Co., Ltd. (Shanghai, China) for sequence determination. The DNA sequences and deduced amino acid sequences were compared with sequences reported in GenBank to confirm the subtypes of the β-lactamase gene.

### Conjugation Experiments and Plasmid Analysis

Sodium azide-resistant *E. coli* J53 was used as a recipient and conjugated to a *bla*_CTX_-_M_-producing isolate by filtration. Transconjugants were selected on Mac Conkey agar containing cefotaxime or ceftazidime (4 μg/ml) and sodium azide (200 μg/ml). ESBL and antibiotic susceptibility was also tested in selected transconjugants, and the presence of *bla* genes was determined using PCR as described above. The resistance plasmids carried by transconjugants were typed using PCR-based replicon typing (42).

### Epidemiological Typing

All the 21 *E. coli* O157:H7 isolates were characterized by pulsed field gel electrophoresis (PFGE) using the CHEF-MAP-PER System (Bio-Rad Laboratories, Hercules, CA, USA) as described by Gautom (43). Briefly, chromosomal DNA of *E. coli* O157:H7 isolates was isolated, and the inserts were digested with *Xba*I (TaKaRa Dalian, China) for 16 h at 37°C. The electrophoresis was performed at 6.0 V/cm for 18.5 h with an angle of 120 at 14°C. The pulse time was increased from 0.5 to 60 s. The *Salmonella* serotype Braenderup H9812 (ATCC BAA-664) was chosen as the molecular weight marker. Gels were then stained in ethidium bromide (1.0 mg/L). Isolates were considered to belong to the same PFGE cluster when the similarity index was >80% (44).

## Results

### Isolation and Presence of Virulence Genes

To investigate the virulence and antibiotic resistance of *E. coli* O157:H7, we collected 2,439 samples from farms and slaughterhouses in Xinjiang regions (Table 2). We successfully isolated *E. coli* clones from all the feces (100%), rectal swabs (100%), and carcass swabs (100%). Studying these *E. coli* isolates, we detected that 21 isolates were the *E. coli* O157:H7 strain (19 isolates collected from cattle farms and 2 isolates obtained from one slaughterhouse). As shown in Table 2, the isolation rates of *E. coli* O157:H7 in feces, rectal swabs, and carcass swabs were 0.7% (8/1155), 1% (11/1236), and 4% (2/48), respectively.

**Table 2:** Sample collection and isolation of *E. coli*.

**Location**	**Source**	**Farm type**	**Sample size and types**	**Numbers and rates (%) in isolation of *E. coli***	**Numbers and rates (%) in isolation of *E. coli* O157:H7**
Akesu	Farms	IST	354 feces	354 (100%)	4 (1%)
Bole	Farms	I	82 rectal swabs	82 (100%)	0
			43 feces	43 (100%)	0
Changji	Farms	IT	211 rectal swabs	211 (100%)	0
			46 feces	46 (100%)	0
Tacheng	Farms	IT	134 feces	134 (100%)	0
Urumqi	Farms	IST	467 rectal swabs	467 (100%)	3 (0.6%)
			90 feces	90 (100%)	0
Wujiaqu	Farms	S	79 rectal swabs	79 (100%)	0
			8 feces	8 (100%)	0
Yili	Farms	IST	480 feces	480 (100%)	4 (0.8%)
			397 rectal swabs	397 (100%)	8 (2%)
	Slaughterhouse		48 carcass swabs	48 (100%)	2 (4%)

*I, industrial farm; S, semi-industrial farm; T, traditional farm*.

Of the 21 *E. coli* O157:H7 isolates, 18 (86%) carried at least one virulence gene and 3 (14%) did not carry any (Table 3). Using PCR technique, we detected that seven (33%) possessed only *stx2*, seven (33%) isolates were positive for *stx1 and stx2*, and only one (5%) isolate had just *stx1* gene. The *eae* gene and *hly* gene were detected in 16 (76%) and 14 (67%) *E. coli* O157:H7 isolates, respectively. *Tccp* in combination with *hly* and *eae* was found in 12 (57%) isolates. In total, six diverse virulence profiles were determined, including *stx1*/*stx2*/*eae*/*hly/tccp* (seven isolates), *stx2*/*eae*/*hly/tccp* (five isolates), *stx2* (two isolates), *eae* (two isolates), *stx1*/*eae*/*hly* (one isolate), and *eae*/*hly* (one isolate) (Table 3).

**Table 3:** Typing antibiotic resistance and virulence genes in *E. coli* O157:H7 isolates.

**Locations**	**Isolates**	**Antibiotic resistance**	**Resistance genes**	**Virulence genes**
Akesu	A1-F1	^−^	^−^	^−^
	A1-F13		^−^	^−^
	A2-F10	^−^	^−^	*stx2, eae, hly, tccp*
	A2-F14	^−^	^−^	^−^
Urumqi	U2-A61-3		^−^	*stx2, eae, hly, tccp*
	U2-A61-4	^−^	^−^	*stx2, eae, hly, tccp*
	U2-A61-5	^−^	^−^	*stx2, eae, hly, tccp*
Yili	Y1-F166	^−^	^−^	*stx1, stx2, eae, hly, tccp*
	Y2-F25	^−^	^−^	*stx2*
	Y2-F27	^−^	^−^	*stx2*
	Y3-F328	^−^	^−^	*stx2, eae, hly, tccp*
	Y4-A20-1	^−^	^−^	*stx1, stx2, eae, hly, tccp*
	Y4-A20-2	^−^	^−^	*stx1, stx2, eae, hly, tccp*
	Y4-A20-3		^−^	*eae*
	Y4-A20-5	^−^	^−^	*eae*
	Y4-A41-2	^−^	^−^	*stx1, stx2, eae, hly, tccp*
	Y4-A41-4	^−^	^−^	*stx1, eae, hly*
	Y4-A103	TET	*tetA*	*eae, hly*
	Y4-A109	AMP, CAZ, CHL, CIP, CTX, LEV, PIP, STR, SXT, TET	*bla*_CTX−M−121_	*stx1, stx2, eae, hly, tccp*
	Y4-C21-1	AMP, CHL, CIP, CTX, LEV PIP, SXT, TET	^−^	s*tx1, stx2, eae, hly, tccp*
	Y4-C21-2	^−^	^−^	*stx1, stx2, eae, hly, tccp*

*Resistance to Ampicillin (AMP), Ceftazidime (CAZ), Chloramphenicol (CHL), Ciprofloxacin (CIP), Cefotaxime (CTX), Levofloxacin (LVE), Piperacillin (PIP), Streptomycin (STR), Trimethoprim-sulfamethoxazole (SXT), Tetracycline (TET). ^−^, undetectable*.

### Antibiotic Resistance Spectrum and Distribution of Antibiotic Resistance Genes

Studying the resistance of isolated *E. coli* O157:H7 to antibiotics, we detected that one isolate (Y4-A103) was resistant to tetracycline and carried the *tetA* gene, which encodes a tetracycline efflux pump. Y4-A109 and Y4-C21-1 were MDR isolates with the resistant patterns: AMP/CAZ/CHL/CIP/CTX/LEV/PIP/SXT/TET (Y4-A109) and AMP/CHL/CIP/CTX/LEV/PIP/SXT/TET (Y4-C21-1). In particular, the Y4-A109 was an ESBL-producing isolate carrying the *bla*_CTX−M−121_ gene (Table 3). Although both Y4-A109 and Y4-C21-1 isolates were resistant to chloramphenicol and sulfonamides, the *cmlAI* and *sulI* genes were not detectable in these isolates, indicating other genes involved in the resistance to chloramphenicol and sulfonamides. In addition, those two MDR isolates (Y4-A109 and Y4-C21-1) simultaneously harbored five virulence genes (*stx1*/*stx2*/*eae*/*hly/tccp*).

### Transferability of *bla*_CTX-M_ Genes and Plasmid Replicon Typing

Studying transferability, we detected that the *bla*_CTX−M−121_ gene of the *E. coli* O157:H7 Y4-A109 isolate was transferable to the recipient strain azide-resistant *E. coli* J53 by conjugation at a frequency of approximately 10^−6^ per donor cell after coincubation of bacteria. We also determined that the resistance of Y4-A109 to ampicillin, cefotaxime, ceftazidime, cotrimoxazole, and tetracycline was also transferable to the recipient. However, plasmid replicon carrying these resistance genes in Y4-A109 remained to be determined.

### Epidemiological Typing

**Figure 1:**
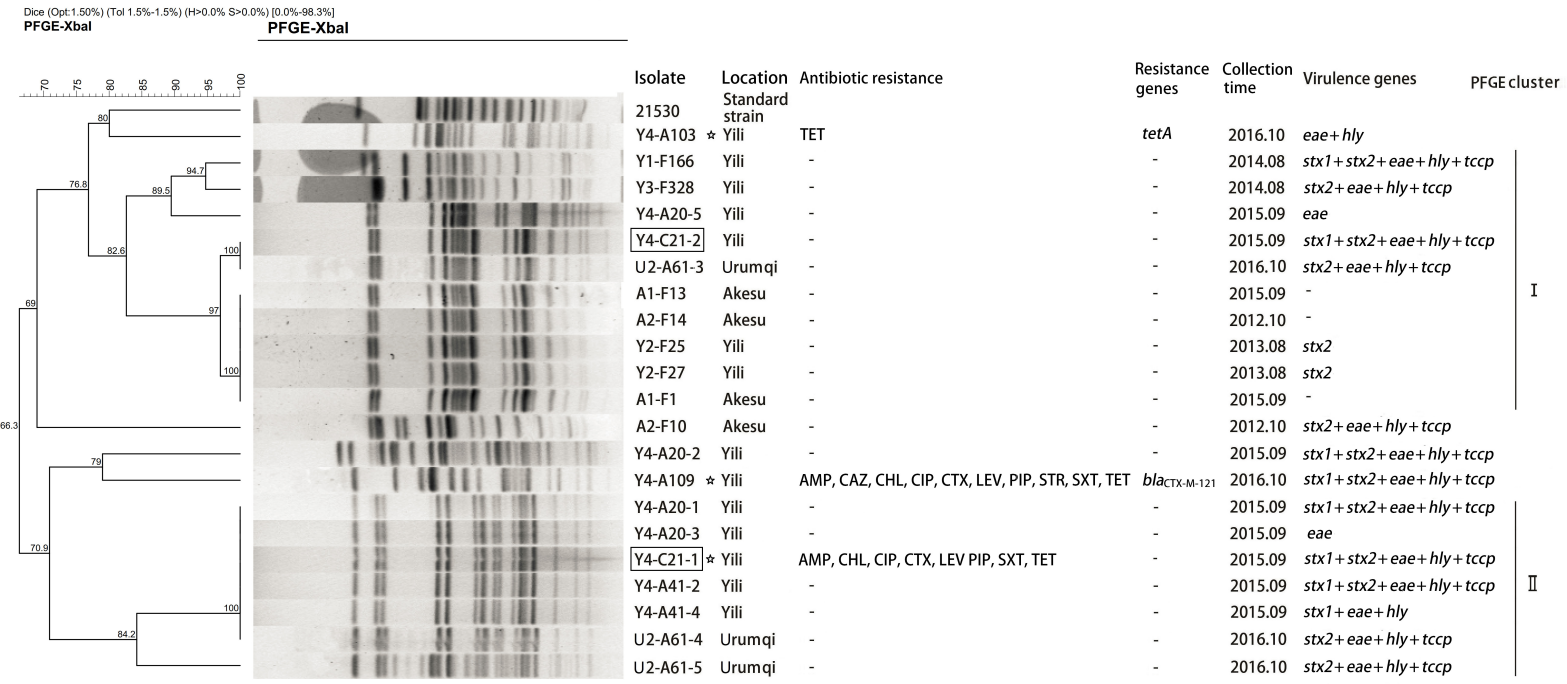
Dendrogram of *Xb*al pulsed-field gel electrophoresis profiles of *E. coli* O157:H7 isolates. The box indicates that the strains isolated from slaughterhouse. The asterisk represents the resistant strains.

Overall, the genetic relatedness ranged from 66 to 100% among the 21 isolates (Figure 1). Furthermore, the studied isolates shared ≤80% genetic similarity to the reference strain 21530. Seventeen of the 21 isolates were grouped into two clusters using >80% similarity of the Dice coefficient. Isolates Y4-A20-1 and Y4-A41-2 (cluster II) were simultaneously isolated from different cattle at Yili in 2015 but shared identical pattern of PFGE, virulence genes, and antibiotic susceptibility (Figure 1). This suggests that potential pathogen transmission might occur from animals to animals within the farm. In addition, three drug-resistant isolates were all identified from Yili. However, they were genetically distantly related (<71% similarity of the Dice coefficient). Noticeably, the PFGE profiles of two isolates from the slaughterhouse were identical to those from the farms. However, Y4-C21-2 carried *stx1* gene, which was absent from its identical farm isolate, whereas Y4-C21-1 appeared to be resistant to eight drugs tested, which were not observed in its identical counterparts (Figure 1).

## Discussion

In this communication, we reported, for the first time, that the *bla*_CTX−M−121_ gene was detected in *E. coli* O157:H7 isolated from cattle in Xinjiang. The *bla*_CTX−M−121_ gene belongs to the *bla*_CTX−M−9_ group. Rao et al. (45) reported the *bla*_CTX−M−121_ gene detected in two *E. coli* isolates collected from farm ducks in China. Zhou et al. (46) identified the *bla*_CTX−M−121_ gene in one *E. coli* isolated from healthy people in Guangdong Province. Jin (47) reported the *bla*_CTX−M−121_ gene in chicken *E. coli* isolated from Guangdong Province. The cephalosporins are used to treat infectious disease such as bovine respiratory infection and mastitis, which may promote production and dissemination of β-lactamase genes (48).

Besides the transferable *bla*_CTX−M−121_ gene between the bacterial strains via plasmid transmission, we also detected a wide spectrum of virulence genes, including the *stx1, stx2, eae, hly*, and *tccp* genes in *E. coli* O157:H7 isolates, which were consistent with the virulence gene types of *E. coli* O157:H7 from bovine in Jiangsu Province (49). We also detected three isolates of *E. coli* O157:H7 lacking any of these virulence genes, which was similar to the bovine *E. coli* O157:H7 isolates reported by Akomoneh et al. (50). *E. coli* O157:H7 isolates possessing only *eae* or *stx2* gene were similar to the isolates obtained from cattle in USA and milk in Nigeria, respectively (51, 52). The attendance of the *eae* gene in O157:H7 STEC (Shiga toxin-producing *E. coli*) isolates resulted in the formation of a highly virulent subpathotype, Enterohemorrhage *E. coli* (EHEC) (53), which was observed in two MDR isolates in the present survey.

In addition, the *tet* resistance gene has been increasingly detected in bovine O157 and non-O157 STEC isolates worldwide (54–56). Our studies also revealed, for the first time, the presence of the *tetA* gene detected in bovine *E. coli* O157:H7 in Xinjiang. Horizontal gene transfer plays a key role in bacterial evolution and transmission of antibiotic resistance genes (57). Resistance traits located in genetic mobile elements, such as plasmids, transposons, and integrons, can be transferred to different strains or bacterial species (58, 59). It is conceivable that virulence gene and drug-resistance gene are carried by the same genetic element; cotransfer may occur under the selection of antibiotics to result in stable virulence clones, thereby leading to production of drug-resistant pathogenic bacteria and persistent bacterial infection in humans and food animals.

We found that the isolates obtained in the same geographical location at the same time had similar PFGE patterns and vice versa, indicating that clonal propagation in cattle population and cross-regional transmission. *E. coli* O157:H7 with identical PFGE pattern (100% similarity) carry different virulence genes and different drug resistance phenotypes, suggesting that the virulence and drug resistance carried by *E. coli* O157:H7 may be acquired or lost during the evolution and transfer of the same cluster of strains. The β-lactam-resistant *E. coli* O157:H7 may give β-lactam resistance to other pathogenic enterobacteria via plasmid-mediated conjugation, thereby posing potential challenges in the management of their associated infectious disease in cattle (60).

*E. coli* O157:H7 was prevalent in 2–15% population of cattle and other animals in China (47). Our results revealed that the overall isolation rate at ~0.9% (21 of 2,439 samples) of *E. coli* O157:H7 and the *bla*_CTX−M_ gene detected in 1 of 21 isolates indicated that the transmission of the *bla*_CTX−M_ gene in *E. coli* O157:H7 population was at an early stage in Xinjiang. Thus, it is important to not only continuously monitor but also identify methods to intervene in the transmission of *bla*_CTX−M_ genotypes to *E. coli* and other bacterial strains, thereby minimizing potential dissemination of β-lactam resistance from the cattle production to their surrounding environment.

## Data Availability Statement

The original contributions presented in the study are included in the article/supplementary material, further inquiries can be directed to the corresponding author/s.

## Ethics Statement

The animal study was reviewed and approved by the Animal Care and Use Committee of Xinjiang Agricultural University. Written informed consent was obtained from the owners for the participation of their animals in this study.

## Author Contributions

ZS and PT conceived and designed the experiments. LZ, MZ, DW, and KM performed the experiments. YZ and YL analyzed the date. PT, LX, and JX contributed to the writing of the manuscript. All authors read and approved the article.

## Conflict of Interest

The authors declare that the research was conducted in the absence of any commercial or financial relationships that could be construed as a potential conflict of interest.
